# Seismic Behavior Analysis of Recycled Aggregate Concrete-Filled Square Steel Tube Frames

**DOI:** 10.3390/ma16124268

**Published:** 2023-06-08

**Authors:** Xianggang Zhang, Xuyan Liu, Yuhui Fan, Junna Yang

**Affiliations:** 1 School of Intelligent Construction, Wuchang University of Technology, Wuhan 430223, China; 2 School of Civil Engineering, Henan Polytechnic University, Jiaozuo 454003, China

**Keywords:** recycled aggregate concrete-filled square steel tube frame, seismic behavior, finite element analysis, hysteretic curve, ductility coefficient, energy dissipation coefficients, stiffness degradation, grey correlation analysis

## Abstract

In this study, the seismic behavior of a recycled aggregate concrete-filled square steel tube (S-RACFST) frame under different design conditions was investigated. Based on previous studies, a finite element model for the seismic behavior of the S-RACFST frame was developed. Moreover, the axial compression ratio, beam–column line stiffness ratio, and yield bending moment ratio of the beam–column were regarded as the variation parameters. It was through these parameters that the seismic behavior of eight S-RACFST frame finite element specimens was discussed. The seismic behavior indexes, such as the hysteretic curve, ductility coefficient, energy dissipation coefficient, and stiffness degradation were obtained—which, in turn, revealed the influence law and the degree of the design parameters regarding seismic behavior. Moreover, the sensitivity of the various parameters with respect to the seismic behavior of the S-RACFST frame was evaluated via grey correlation analysis. The results show that the hysteretic curves of the specimens were fusiform and full with respect to the different parameters. Firstly, with the axial compression ratio increasing from 0.2 to 0.4, the ductility coefficient increased by 28.5%. In addition, the equivalent viscous damping coefficient of the specimen with the axial compression ratio of 0.4 was 17.9% higher than that of the specimen with the axial compression ratio of 0.2, which was 11.5% as well as that with an axial compression ratio of 0.3. Second, when the line stiffness ratio rises from 0.31 to 0.41, the specimens’ bearing capacity and displacement ductility coefficient both get better. However, the displacement ductility coefficient gradually decreases when the line stiffness ratio is greater than 0.41. As a result, an optimal line stiffness ratio (0.41) thus exhibits good energy dissipation capacity. Thirdly, with the increase in the yield bending moment ratio from 0.10 to 0.31, the bearing capacity of the specimens improves. In addition, the positive and negative peak loads increased by 16.4% and 22.8%, respectively. Moreover, the ductility coefficients were all close to three, thus demonstrating good seismic behavior. The stiffness curve of the specimen with a large yield bending moment ratio with respect to the beam–column, is higher than those that possess a small beam–column yield moment ratio. In addition, the yield bending moment ratio of the beam–column possesses a significant influence on the seismic behavior of the S-RACFST frame. Furthermore, the yield bending moment ratio of the beam–column should be considered first in order to ensure the seismic behavior of the S-RACFST frame.

## 1. Introduction

With the rapid development of the construction industry, natural aggregates were, and continue to be, consumed in large quantities. At the same time, the construction process produced a great deal of construction waste, thus leading to severe environmental pollution [1,2,3]. The crushing, cleaning, and processing of construction waste in order to produce recycled aggregate concrete (RAC) can contribute toward significant benefits [4,5,6]. However, compared with ordinary concrete, recycled aggregate concrete possesses certain flaws, such as low strength and a significant degree of water absorption [7,8,9]. With respect to this, steel tubes inhibit the generation and development of vertical cracks in concrete, thus the restraining quality of steel tubes can also improve the compressive strength and deformation ability of concrete [10,11,12,13]. The local buckling of the steel tube may be successfully avoided by the support effect of RAC deformation on the interior of the steel tube during the application of stress. Therefore, the recycled aggregate concrete-filled steel tube (RACFST) can utilize the benefits of steel tubes and RAC for the purposes of increasing force characteristics, as well as with respect to addressing the issue of construction waste. In so doing this, a wide range of application possibilities in the growth of the green building industry is opened up.

In order to promote the application of the RACFST in the context of practical engineering, it is necessary to reveal the earthquake failure mechanism of RACFST members and frames. Certain scholars demonstrated the difference in seismic behavior failure mechanisms between the RACFST columns and with ordinary concrete-filled steel tube columns. Moreover, Tang et al. [14,15] conducted the cyclic loading test on the RAC and on ordinary concrete-filled steel tube columns, then studied their respective seismic behaviors. The RACFST column exhibited a superior bearing capacity and ductility, as well as, at the same displacement level, a slightly lower energy dissipation coefficient. The research shows that the RACFST column possesses excellent seismic performance and provides a reference for the theoretical research of the RACFST. However, the research was only carried out with respect to the concrete-filled steel tube column, which incorporated 100% recycled concrete aggregates and ordinary concrete-filled steel tubes. This approach, however, neglected to account for the fact that the shape of the steel tube and the replacement ratio of the recycled aggregate can also affect the performance of the RACFST column. Therefore, certain scholars took a closer look in regard to this. Yang et al. [16] studied the effect regarding the shape of the steel tube, as well as with respect to the replacement ratio of the recycled concrete on the seismic behavior of the RACFST. The failure mode of the RACFST column is similar to that of an ordinary concrete-filled steel tube column. The ultimate bearing capacity of the circular RACFST column, with a 25% replacement rate, is lower than that of the square RACFST column, whereby it is composed of a 50% recycled coarse aggregate, which is higher than that of the square RACFST column. Xu et al. [17] studied the seismic behavior of the square RACFST columns by changing the replacement ratio of the RAC and then established a new calculation method for the bearing capacity of the RACFST. The results show that changing the replacement ratio of the recycled aggregate has little effect on the hysteretic properties of the RACFST column. Moreover, the displacement ductility can still meet the seismic requirements. Due to the above studies being different in terms of the source and particle size of the recycled aggregate, loading device, and the selection of design parameters, there may be some minor errors in the test results. However, the conclusion is the same: the seismic behavior of the RACFST column undergoes little change with respect to the increase in the replacement ratio of the recycled aggregate. Indeed, the hysteretic performance of the RACFST column is similar, or slightly altered, with respect to that of ordinary concrete-filled steel tube columns; in addition, the seismic performance remains good. With respect to this, engineering bearing structures can use RACFST columns based on their seismic performance index. In order to study the seismic behavior of recycled aggregate concrete frame joints, Zhou et al. [18] discussed the influence of length and volume content of the fiber that are regarded as the parameters of RAC joints. In addition, Su et al. [19] took the replacement ratio of recycled aggregate as the change parameter in order to carry out a cyclic loading test on the recycled concrete frame joints. Subsequently, Xiao et al. [20] adopted a recycled coarse aggregate as the structural material in a novel beam–column joint with design for deconstruction connections. The above studies demonstrate that the RAC is feasible in the practical application of frame joints, but this does not extend to the finite element model in regard to successfully analyzing the performance of recycled concrete frame joints under different parameters. The RACFST, therefore, is generally utilized as columns or arch ribs for the purposes of axial load-bearing structures. The other components and overall structural form affect the force characteristics of the columns and the arch ribs in the structure. Therefore, their mechanical properties and working mechanisms differ from the individual components. As such, the overall structural performance requires more intensive study, especially in regard to the seismic behavior of the overall frame structures. 

Combined with the current body of research regarding steel tube recycled concrete columns and recycled aggregate concrete frame joints, Wen et al. [21] discussed the experimental research on the seismic behavior of steel tube, bulk mass, recycled, and concrete column–steel beam frameworks. They simulated the finite element simulation based on the test, but the number of variable parameters and the analysis depth of the finite element simulation required increasing. In addition, more details regarding the numerical modeling did not provide much with respect to readership and remodeling. Moreover, they used large-size recycled concrete blocks, which have a positive impact on the popularization and utilization of discarded concrete. However, the existence of blocks reduces the bearing capacity and hysteretic properties of specimens when compared with all cast-in-place columns. Regarding cast-in-place concrete, Chen et al. [22] researched the seismic behavior of the RACFST circular frames. The frames demonstrated optimal deformation performance, collapse resistance, and energy dissipation capacity. In addition, the frame possessed exemplary seismic behavior, which could be applied in high-rise buildings in order to mitigate the dangers that occur in high-intensity seismic areas. However, both studies only discussed the seismic behavior of the RACFST circular frames. Our group [23], instead, conducted small-scale model tests on recycled concrete frames with square steel tubes. The ductility of the S-RACFST was found to be higher than that of the circular specimen reported by Chen et al. [22]. However, this study mainly focuses on the test, whereas there are few other studies that investigated the variation parameters.

To summarize, combining the advantages of renewable resources and concrete-filled steel tubes can solve environmental problems and can also still possess good seismic performance. Having said this, a study on the seismic behavior of the RACFST frame still needs to be perfected. However, relying entirely on a test for this aspect is too expensive, and it is difficult to establish a complete research system utilizing it alone. As such, it is essential to establish an accurate finite element model for the purposes of parametric analysis. In this study, as based on the group’s previous studies [23], the seismic behavior of the RACFST frame was numerically analyzed. Indeed, the finite element model of the S-RACFST frame was established by ABAQUS software, and the design parameters of the specimen were changed in order to obtain the seismic behavior indicators, such as the hysteretic curve, ductility coefficient, energy dissipation coefficient, and stiffness degradation. The sensitivity of each parameter to the seismic behavior of the specimen was evaluated via grey correlation analysis, which provides a reference for the engineering design and theoretical analyses.

## 2. Establishment of the Finite Element Model for the Seismic Behavior of the S-RACFST Frame Specimens

### 2.1. Design of Specimens

**Figure 1:**
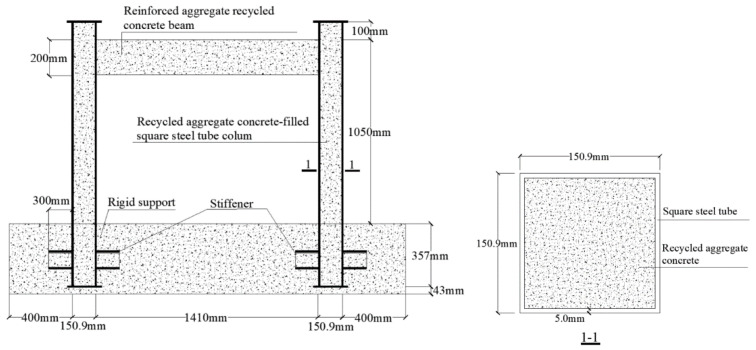
S-RACFST frame specimen.

The S-RACFST frame seismic test model that was constructed and completed in the group’s previous study [23] was selected for the current work. The frame structure geometry of the model is shown in Figure 1. The applied coarse aggregates were RCAs obtained from the waste concrete specimens in the Key Laboratory of Disaster Prevention and Structural Safety of China, Ministry of Education. The physical properties of the RACs are provided in Table 1. The strength grade of the RAC in regard to the steel tube and beam was C40; the replacement ratio of the recycled coarse aggregate was 100%; the physical properties of the recycled aggregate and the steel tube grade was Q235; and the test axial compression ratio was taken as 0.8. The frame-specific design parameters, reinforcement, and material properties were reported, in detail, in a previous study by the group [23].

### 2.2. Loading System

In the actual project, the situation of the frame was simulated in this study. The same vertical loading was applied simultaneously to the top of the two columns. Then, a low-frequency cyclic lateral load was applied transversely and horizontally when the vertical loading stabilized. The 1500 kN hydraulic jack was used to apply the vertical loading at the top of the column; further, this loading was maintained throughout the loading operation. In the process of horizontal loading, joint control of force and displacement was used. In addition, when conducting the horizontal loading, a graduated approach with 5 kN loading levels was used, with each loading level cycled once before the specimen yields. The displacement loading was carried out in multiples of the yield displacement Δ_y_ after the specimen yields were performed, thereby corresponding to three cycles per loading step. The loading was stopped when the loading dropped to 85% of the peak loading. Lastly, the model was cycled once under each loading level during the validation and extended analysis stage in order to save time and cost.

### 2.3. Material Constitutive Model

The constitutive behavior of the steel-adopted elastic–plastic hardening model was determined, including with respect to the elastic and intensive sections. This constitutive behavior not only accurately simulates the properties of steel but also facilitates the convergence of the finite element model. After the yield stress is reached in the elastic phase σ=Es⋅ε, the plastic modulus of the steel is taken as σ=0.01Es in the intensive section. In addition, where σ is the stress, *E*_s_ is the elastic modulus of steel, and *ε* is the strain.

The RAC was analyzed within a plastic damage model; moreover, the corresponding key parameters for this are shown in Table 2. The concrete damage factors, in regard to tension and compression, are calculated as in Equation (1), according to the energy equivalence principle [24].

(1) $$d=1-\sqrt{\frac{\sigma }{E_{0}\varepsilon }}$$

where *d* is the concrete damage factor; *σ* is the real concrete stress; *ε* is the concrete strain; and *E*_0_ is the initial concrete modulus of elasticity.

The compressed constitutive relationship of the interior RAC in the S-RACFST was detailed in the previous research results of the authors [25]. However, the compressed constitutive relationship of the RAC in the beam was adopted from Xiao [26]. The constitutive relationship considers the effect of the replacement ratio of the RAC, which was based on the ordinary concrete in the “Code for design of concrete structures” (China GB50010-2010). The constitutive relationship expressions are shown in Equations (2)–(6).

(2) $$y=ax+\left(3-2a\right)x^{2}+{\left(a-2\right)x^{3}}_{\;(0\;⩽\;x\;⩽\;1)}$$

(3) $${y=\frac{x}{b{\left(x-1\right)}^{2}+x}}_{\;(x\;>\;1)}$$

where

(4) $$a=2.2\left(0.748R^{2}-1.231R+0.975\right)$$

(5) $$b=0.8\left(7.6483R+1.142\right)$$

(6) $$x=\frac{\varepsilon }{\varepsilon_{c}},\;y=\frac{\sigma }{f_{c}}$$

$\varepsilon_{c}$ is the RAC peak strain, *R* is the replacement ratio of RA, and *f*_c_ is the measured RAC compressive strength.

As the tensile performance of the interior RAC is not significantly affected by the restraint of the external steel tube, the uniaxial tensile stress–strain relationship of the interior RAC is taken according to the “Code for design of concrete structures” (China GB50010-2010) as shown in Equations (7) and (8).

(7) $$y=\left\{\begin{array}{ll} 1.2x-0.2x^{6} & \left(\varepsilon \leq \varepsilon_{p}\right)\\ \frac{x}{0.31\sigma_{p}{\left(x-1\right)}^{1.7}+x} & \left(\varepsilon >\varepsilon_{p}\right) \end{array}\right.$$

where

(8) $$x=\frac{\varepsilon_{c}}{\varepsilon_{p}},\;y=\frac{\sigma_{c}}{\sigma_{p}}$$

$\sigma_{p}$ is the peak tensile stress, $\sigma_{p}=0.26{\left(1.25f_{c,R}^{\prime }\right)}^{2/3}$, $\varepsilon_{p}$ is the peak tensile strain, and $\varepsilon_{p}=43.1\sigma_{p}(\mu \varepsilon )$.

### 2.4. Modeling Method

The interaction between the square steel tube and the interior RAC adopted “surface-to-surface contact”. Further, a small bond slip could occur between the square steel tube and the RAC. The normal direction model adopted the “hard” contact, and the tangent direction model adopted the “penalty” contact. In addition, the friction coefficient was taken as 0.25 [27]. The reinforcement skeleton was embedded in the RAC beam by “embed”, whereby the effect of the bonding slip was neglected. The steel tube and RAC were all made of the same 8-node hexahedral linear reduced integral unit (C3D8R), and the three-dimensional two-node truss unit (T3D2) was used for the reinforcing bars and stirrups in the beam.

**Figure 2:**
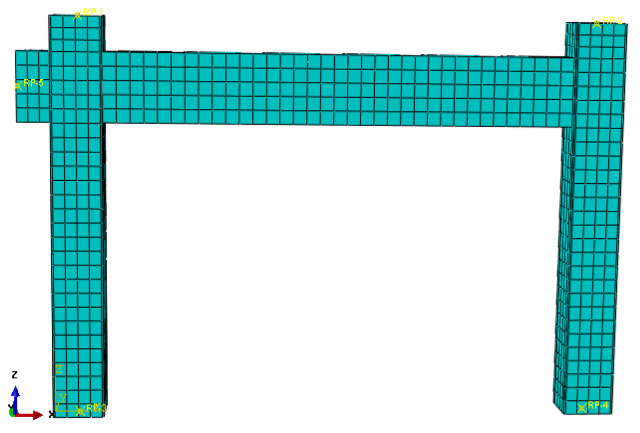
Mesh of the S-RACFST frame specimen.

**Figure 3:**
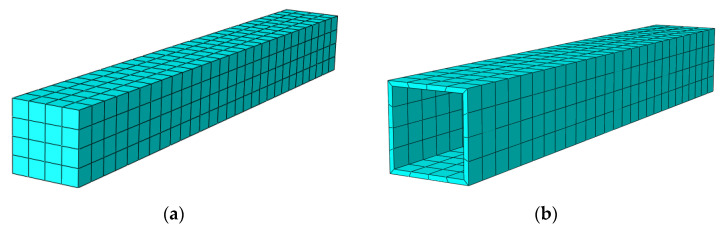
The meshes of the components: (**a**) Interior RAC in the square steel tube; (**b**) The square steel tube component.

The mesh division density is very crucial for the finite element model analysis calculation. First, with respect to the initial analysis, a reasonable mesh division was taken. Then, the mesh density division was doubly expanded and then analyzed again. Next, the two results were compared until the two differences were found to be less than 1%. Then, the size of 40 mm was finally chosen. The mesh division in regard to the finite element model of the S-RACFST frame specimen is shown in Figure 2. Further, the mesh division of the interior RAC and the square steel tube components are shown in Figure 3.

The boundary condition of the finite element model was consistent with the test that was established according to the test conditions. The bottom of the finite element model steel tube was completely fixed—i.e., *U*_x_ = *U*_y_ = *U*_z_ = *UR*_x_ = *UR*_y_ = *UR*_z_ = 0—whereby the vertical pressure was applied to the top of the steel tube along the *Z*-axis, and a low-frequency cyclic lateral displacement loading was applied along the horizontal *X*-axis. Then, two analysis steps in Abaqus were constructed in order to apply the loading. The boundary conditions and vertical loading were applied in the first analysis step. In the second analysis step, the horizontal loading was controlled by the displacement that was applied to the joints. The same displacement amplitude as the actual test was entered in the “Amplitude” command. Furthermore, the S-RACFST frame specimen displacement control was applied through a reference point that was coupled to a horizontal loading mat plate.

Due to the incremental–interactive method possessing the advantages of the incremental and iterative methods, the incremental–interactive method was selected for the calculation in this study. In addition, the automatic incremental method was used for the incremental method. Regarding the iterative method, Newton’s method is computationally large and the convergence is good; thus, Newton’s method was chosen in this study.

### 2.5. Finite Element Model Validation

**Figure 4:**
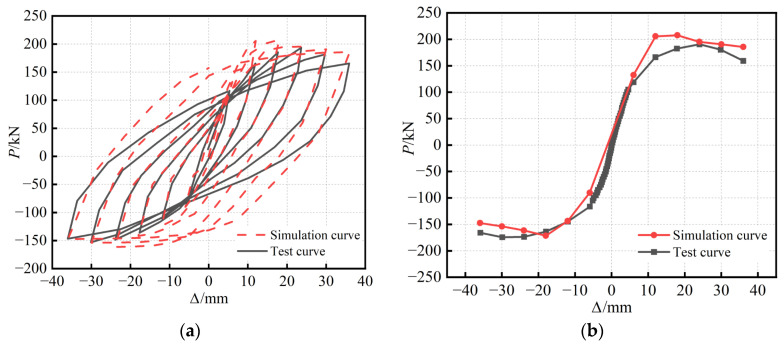
Comparison of the S-RACFST frame specimen simulation and test: (**a**) Hysteretic curve; (**b**) Backbone curve.

Figure 4a shows the results of the finite element hysteretic simulation curve for the S-RACFST frame specimen when compared with the test. The hysteretic test curve is derived from the previous research results of our group [23]. In addition, it is shown that the hysteretic curve obtained from the simulation generally agrees well with the test. The simulation curves of the elastic phase during the positive and negative loading process agree with the test curves. Furthermore, the hysteretic simulation curve in the elastic–plastic stage exhibits a higher loading stiffness than the hysteretic test curve. Moreover, the hysteretic loop area obtained from the finite element is slightly larger than the test. The difference between the hysteretic loop areas is relatively more pronounced in the later loading period. The reason for this is that the geometric defects, concrete cracks, and damage during the test are not fully considered in the simulation analysis. Therefore, the bearing capacity and hysteresis loop area obtained from the simulation are higher than those found in the test. Overall, the model can simulate the whole process of specimen development under reciprocal loading.

Figure 4b shows the results of the finite element simulative backbone curve for the S-RACFST frame specimen when compared with the experiment. The stiffness of the finite element model is slightly more significant than the test in the early loading stage. The descending section of the backbone curve for the positive simulation is slightly slower than the test curve. Furthermore, the descending section of the negative simulative backbone curve is slightly steeper than the test curve. The reason for this is the difference between the adopted constitutive behavior and the actual material properties of the steel, as well as the fact that the concrete damage is not considered accurate enough. Overall, the experimental results are in good accordance with the simulative results.

The peak point of the backbone curve of the S-RACFST frame specimen regarding the simulation and test is shown in Table 3. The difference between the finite element calculated peak load and the test peak load was found to be minuscule. The finite element calculated peak displacement could be used in order to estimate the test peak displacement. Based on the verification of the hysteretic curve and the backbone curve, the finite element model established in this study possesses high computational accuracy and can meet the requirements of subsequent analysis.

## 3. Design of the Finite Element Specimen

Eight parametric analysis models for the specimen were established as the various parameters based on the finite element model, axial compression ratio (*n*), beam–column line stiffness ratio (*i*), and yield bending moment ratio of the beam–column (*k*_m_), as shown in Table 4. The concrete strength grade of the specimens was C40. In addition, by taking Nx-Ix-Kx as an example for the naming of the specimens, N1~N4, respectively, indicate the *n* of 0.36, 0.20, 0.30, and 0.40; I1 ~ I3, respectively, indicate the (*i*) of 0.62, 0.41, and 0.31; and K1~K3, respectively, indicate the (*k*_m_) of 0.10, 0.19, and 0.31.

## 4. Results and Analysis

### 4.1. Hysteretic Curves

**Figure 5:**
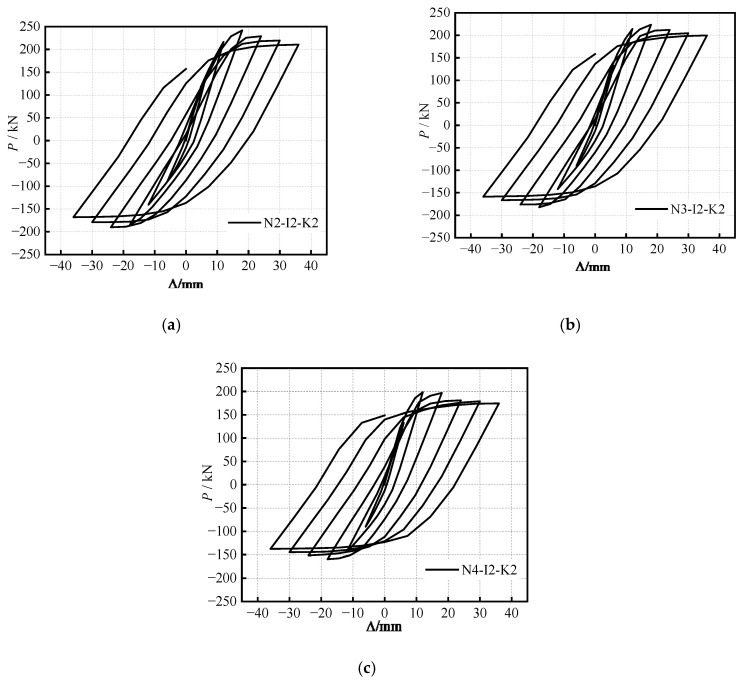
Hysteretic curves of specimens under different (*n*): (**a**) *n* = 0.2; (**b**) *n* = 0.3; (**c**) *n* = 0.4.

The hysteretic curves of the S-RACFST frame specimens under the different (*n*) are shown in Figure 5. The hysteretic curves of the specimens at the same (*n*) are observed to be similar, thereby exhibiting the fusiform, as well as the smooth and full behavior, which indicates a strong energy dissipation capacity with respect to the specimens. The hysteretic curve becomes full, and the energy dissipation capacity gradually increases after 2Δ_y_ (Δ_y_ represents displacement at the yield point) with the enhancement of (*n*), whereas the peak bearing capacity gradually decreases.

**Figure 6:**
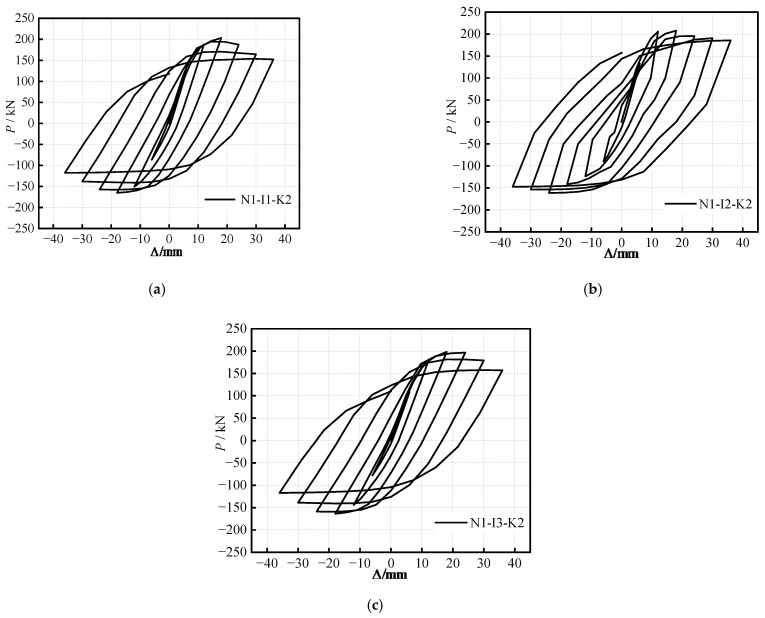
The hysteretic curves of specimens under different (*i*): (**a**) *i =* 0.62; (**b**) *i =* 0.41; (**c**) *i =* 0.31.

The hysteretic curves of the S-RACFST frame specimens under the different (*i*) are shown in Figure 6. When comparing the specimens with different (*i*), the shape of the hysteretic curves is observed to be similar, with each curve exhibiting a full fusiform shape. Thus, the specimens exhibit an ideal energy dissipation behavior. The bearing capacity of the specimens is gradually reduced with the subsequent decrease in (*i*). The slope of the curve for each specimen is observed to decrease gradually with increasing cyclic displacement—in other words, the smaller the (*i*) of the S-RACFST frame specimen, the lower the energy dissipation capacity.

**Figure 7:**
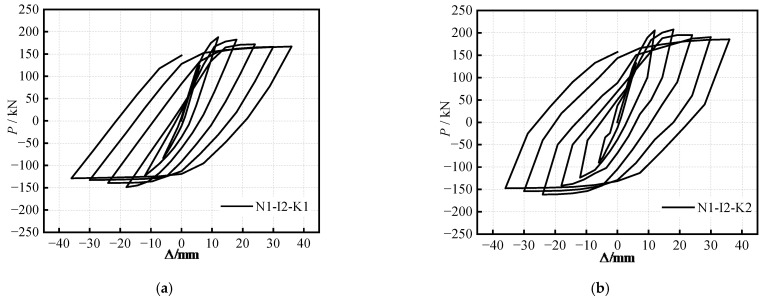

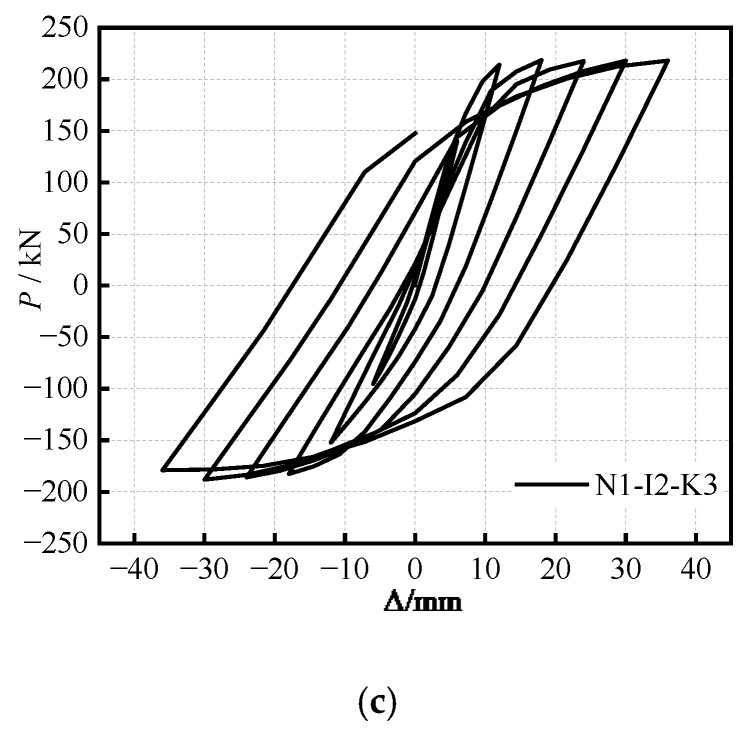
The hysteretic curves of specimens with different (*k*_m_): (**a**) *k*_m_ = 0.10; (**b**) *k*_m_ = 0.19; (**c**) *k*_m_ = 0.31.

The hysteretic curves of the S-RACFST frame specimens under different (*k*_m_) are shown in Figure 7. The hysteretic curves of the S-RACFST frame specimens are observed with respect to the fusiform and full shape, thereby exhibiting exemplary seismic behavior and energy dissipation capacity. The peak bearing capacity is significantly increased with enhancing (*k*_m_), and the specimens exhibit superior seismic behavior.

### 4.2. Ductility Coefficient

**Figure 8:**
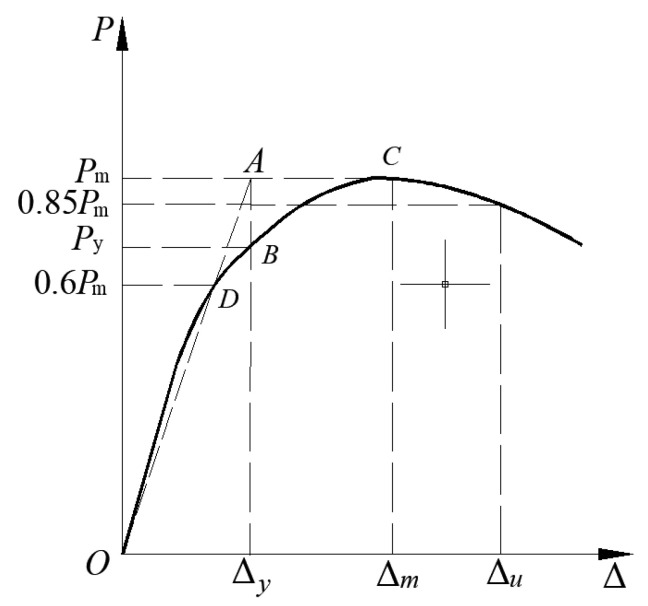
The schematic diagram of the yield point determination for the S-RACFST frame specimen [22].

The S-RACFST frame specimen’s yield point could be determined via using the graphical method as reported by Chen et al. [22], as shown in Figure 8. With respect to the load–displacement curve, the loading value corresponding to 0.6 *P*_m_ was determined and used in order to construct a straight line that was parallel to the horizontal axis, i.e., the line intersecting the load–displacement curve at point *D*. Subsequently, a diagonal line was drawn through the origin, and the horizontal line of the peak loading at point *A* was extended. The load–displacement at point *B* was then crossed as the vertical line of the abscissa through point *A*. Moreover, *B* was marked as the equivalent yield point, and the constant load and displacement were termed the yield load *P*_y_ and the yield displacement Δ_y_. The failure point was the loading value (0.85 *P*_m_) and the limit displacement (Δ_u_) of the corresponding point as the peak loading dropped to 85%. 

The displacement ductility coefficients, the characteristic point load, and the displacement of the S-RACFST frame specimens under different (*n*) are shown in Table 5. Furthermore, the yield displacement and the load exhibit a decreasing trend with the subsequent increase in (*n*). As the (*n*) increases from 0.2 to 0.4, the yield load decreases by 23.9%, whereas the yield displacement decreases from 12.66 mm to 9.77 mm. This, therefore, indicates that the (*n*) significantly influences the yield point of the specimens. At the (*n*) of 0.2, the corresponding ductility coefficient average value is determined to be 2.88. On the other hand, when the (*n*) is 0.4, the corresponding ductility coefficient average value is noted to be 3.70. Thus, as the (*n*) is enhanced from 0.2 to 0.4, the average displacement ductility coefficient increases by 22.2%. The displacement ductility coefficient increases with the subsequent increase in (*n*), which may be why the restraint square steel tube that is imposed on RAC is enhanced, thus improving the whole deformation capacity of the specimen with the increase in (*n*).

The displacement ductility coefficients, the characteristic point load, and the displacement of the S-RACFST frame specimens with different (*i*) are shown in Table 6. First, the displacement ductility coefficient is observed to increase; then, a reduction with the increase in (*i*) is observed. The displacement ductility coefficient of the specimens under different (*i*) is noted to be higher than 2.5, which meets the seismic behavior requirement with respect to the frame structure. Indeed, the ductility coefficient was 3.39, and the displacement ductility coefficient was 23%~25% higher than that of the other two specimens when (*i*) is 0.41. This is because the appropriate (*i*) enables the beam to transmit horizontal force better, such that the beam and column can work well together. Therefore, an optimal (*i*) can improve the ductility of the S-RACFST frame specimen.

The displacement ductility coefficient, the characteristic point load, and the displacement of the specimens with different (*k*_m_) are shown in Table 7. It can be seen that the peak bearing capacity of specimens was improved, as well as the fact that the positive and negative peak loadings increase by 16.4% and 22.8% with the increase in (*k*_m_) from 0.10 to 0.31, respectively. However, the displacement ductility coefficient decreases accordingly. At the (*k*_m_) of 0.10, 0.19, and 0.31, the ductility coefficient varies by −3.69% and −5.01%, respectively. Therefore, the change in (*k*_m_) significantly influences the frame’s bearing capacity. Moreover, the ductility coefficient is somewhat reduced but still greater than 3, thereby showing good seismic ductility. In addition, the S-RACFST frame structure can meet the requirements of the ductility structure with different (*k*_m_).

### 4.3. Energy Dissipation Capacity

**Figure 9:**
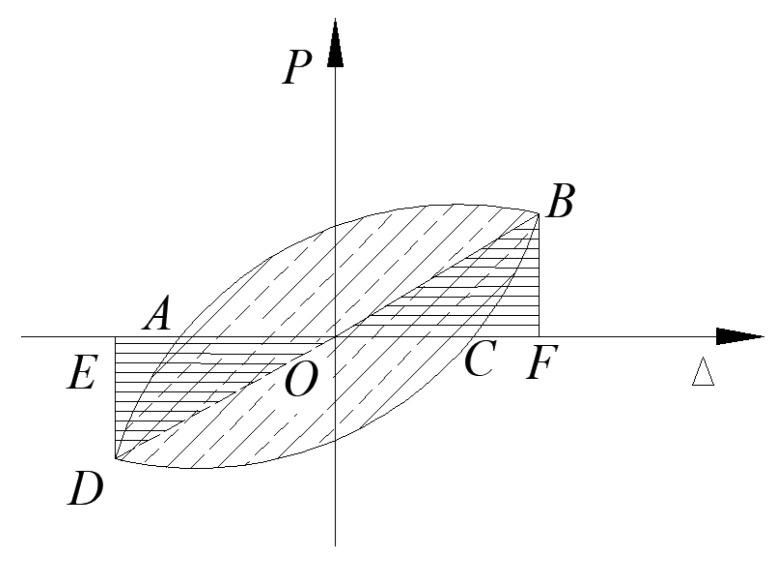
The hysteresis loop of load deformation [22].

The energy dissipation capacity is a considerable index with respect to the analysis of the seismic behavior of the specimens. In this study, the equivalent viscous damping coefficient *h*_e_ was measured in terms of the elastic–plastic energy dissipation capacity of the S-RACFST frame specimens. Furthermore, the calculation method of *h*_e_ was used and followed, as reported by Chen et al. [22].

(9) $$h_{e}=\frac{S_{ABCD}}{2\pi \cdot (S_{BOF}+S_{AOD})}$$

where *S_ABCD_* represents the envelope area of a complete hysteresis loop. In addition, *S_BOF_* + *S_AOD_* represents the sum of the areas of the triangles consistent with the upper and lower climaxes of the specimen loading hysteresis loop, as shown in Figure 9.

The *h*_e_ value of the S-RACFST frame specimens for the different (*n*) is shown in Table 8. The *h*_e_ value of each specimen was noted to increase gradually with each increasing cyclic displacement. At the end of loading, the *h*_e_ of the specimen reached 0.3, thereby indicating that the specimen possessed good energy dissipation capacity. Indeed, the *h*_e_ of the specimen with (*n*) of 0.4 reaches 0.348, 17.9%, and 11.5% higher than that of the specimen with (*n*) of 0.2 and 0.3. The *h*_e_ value was observed to be above 0.3, which indicates an optimal energy dissipation capacity. Regarding the specimens for different (*n*), the *h*_e_ value of the specimens under a more significant (*n*) were noted to be higher at the same displacement. The *h*_e_ of the characteristic points for specimens under different (*n*) are shown in Table 9. As the specimen yielded, the *h*_e_ value of the specimen with the (*n*) of 0.2 was greater than that of the specimens with (*n*) of 0.3 and 0.4. The *h*_e_ value of the peak and failure point for the specimen with the (*n*) of 0.4 was noted to be higher than those of the other two specimens with enhancing cyclic displacement. Therefore, the larger the (*n*), the superior the energy consumption performance of the S-RACFST frame specimens.

The *h*_e_ value of the specimens for different (*i*) are shown in Table 10. With respect to enhancing the cyclic displacement, the *h*_e_ value of the specimens for the same (*i*) gradually increases and, subsequently, the energy consumption capacity increases. Once the loading ends, the *h*_e_ value of the specimen with a large (*i*) is noted to be significantly higher than that of the specimen with a small (*i*). With respect to the (*i*) of the 0.62 specimen, the *h*_e_ was 0.381, which was 7.6% higher than the *h*_e_ of the 0.31 specimen. The reason for this was due to the fact that the restraint capacity of the beam to the column increases with the subsequent increase in (*i*). As such, the cracking damage of the recycled concrete in the column decreases, and the collective deformation capacity between the beam and column is thus strengthened. The *h*_e_ of the characteristic points for the specimens under different (*i*) are shown in Table 11. The *h*_e_ of the specimens was noted to be greater than 0.26 with different (*i*) when at failure, which is bigger than the equivalent viscous damping coefficient (0.1 < *h*_e_ < 0.2) [28] in regard to the conventional reinforced concrete structure. Therefore, the energy dissipation capacities of the S-RACFST frame specimens under different (*i*) were found to be superior.

The *h*_e_ value of the specimens with different (*k*_m_) are shown in Table 12. The equivalent, viscous damping coefficient of the specimens gradually enhanced with the increase in cyclic displacement along with the amplitude of the increment, due to the continuous presence of the plastic hinges at the beam end. As a result, an increasing extent of energy was absorbed when the structure entered the elastic–plastic stage. In addition, the specimens exhibit an excellent energy consumption capacity. Furthermore, the *h*_e_ of the characteristic points for the specimens under different (*k*_m_) are shown in Table 13. The *h*_e_ value of the specimens with different (*k*_m_) are not significant at failure, thereby indicating that the (*k*_m_) possesses an insignificant effect on the energy dissipation capacity of the characteristic points in the S-RACFST frame specimens.

### 4.4. Stiffness Degradation

In this study, the secant stiffness reflected the changes in the specimens under repeated loading. The formula for calculating *K_i_* can be found in the Chinese Specification JGJ/T 101-2015 as

(10) $$K_{i}=\frac{\left|+F_{i}\right|+\left|-F_{i}\right|}{\left|+X_{i}\right|+\left|-X_{i}\right|}$$

where +*F_i_* represents the positive peak load under the *i*-th loading level; −*F_i_* represents the negative peak loading under the *i*-th loading level; +*X_i_* represents the positive amplitude under the *i*-th loading level; and −*X_i_* is the negative amplitude under the *i*-th loading level.

**Figure 10:**
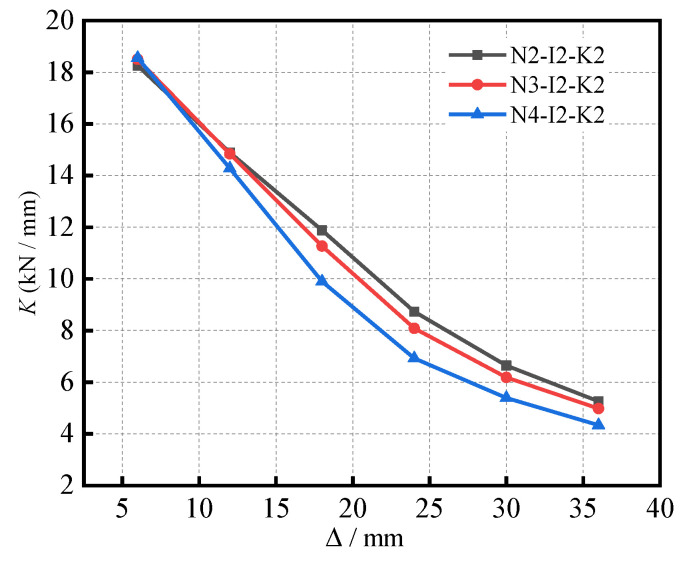
The stiffness degradation curves of the specimens under different (*n*).

The stiffness degradation curves of the specimens under different (*n*) are shown in Figure 10. Additionally, the specimens exhibit a gradual stiffness degradation for the different (*n*). At the (*n*) of 0.2, the initial stiffness of the specimen was observed to be 18.26 kN/mm. At the (*n*) of 0.3, the initial stiffness of the specimen was observed to be 18.50 kN/mm. Likewise, at the (*n*) of 0.4, the initial stiffness of the specimen was 18.56 kN/mm. As the (*n*) increased from 0.2 to 0.4, the initial stiffness of the specimens was noted to be similar. With a gradual increment in the load and displacement, the cracks in the specimens continue to generate and expand; further, the stiffness demonstrates a downward trend. From the initial point to the peak point, the stiffness decreased rapidly. After reaching the peak loading, the stiffness degradation rate decreased, and the curves tended to become flat. In general, the stiffness of the specimens with different (*n*) tended to decrease with increasing displacement, thus revealing a rapid decline in the beginning, as well as a gradual plateauing.

**Figure 11:**
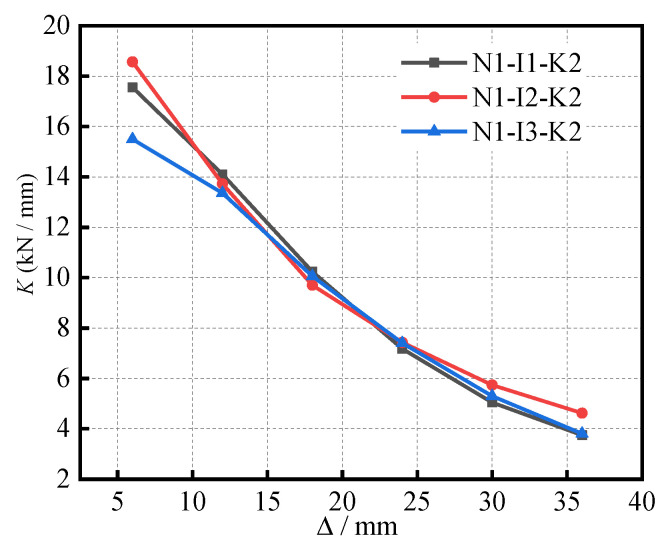
The stiffness degradation curves of the specimens under different (*i*).

The stiffness degradation curves of the S-RACFST frame specimens under different (*i*) are shown in Figure 11. With respect to the (*i*) of 0.62, 0.41, and 0.31, the initial stiffness of the specimens was determined to be 17.55 kN/mm, 18.57 kN/mm, and 15.50 kN/mm, respectively. Thus, with respect to the (*i*) of 0.31, 0.41, and 0.62, the initial stiffness of the specimens was changed by 19.8% and −5.49%, respectively. The reason for this was the fact that the secant stiffness of the specimens deteriorated rapidly with the cracks of recycled concrete beams and the cracking of RAC inside the steel tube. In the late loading period, the cracks of the RAC beams were complete, the plastic hinge of the column bottom gradually formed, and the stiffness degradation rate of the specimens gradually slowed down and tended to be consistent.

**Figure 12:**
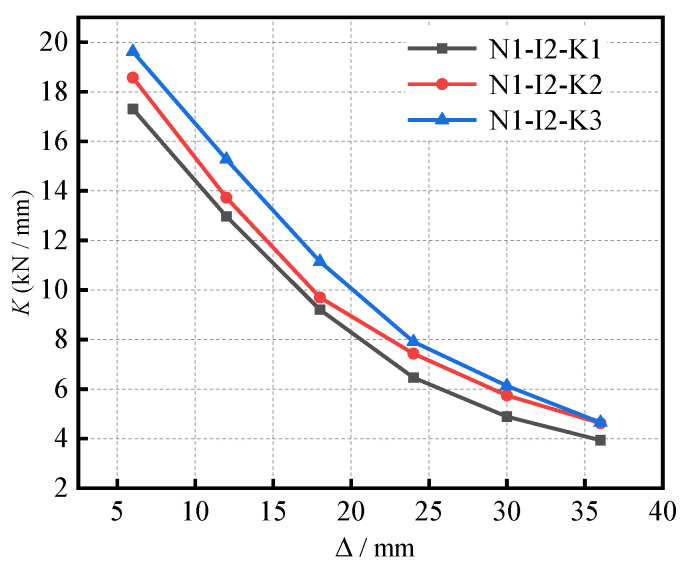
The stiffness degradation curves of specimens under different (*k*_m_).

The stiffness degradation curves of the S-RACFST frame specimens under different (*k*_m_) are shown in Figure 12. Moreover, the initial stiffness of the specimens was 17.30 kN/mm, 18.57 kN/mm, and 19.62 kN/mm, whereby the different values of (*k*_m_) were 0.10, 0.19, and 0.31, respectively. Moreover, the initial stiffness of specimens varied between 7.34% and 5.65% when the beam–column linear stiffness ratio was 0.10, 0.19, and 0.31, respectively. During the early loading stage, the cracks began to appear continuously. With a gradual increment in the load and displacement, the cracks in the specimens continued to expand. Furthermore, the stiffness of the specimens exhibited a declining tendency. From the initial point to the peak point, the stiffness was noted to rapidly reduce. Moreover, the stiffness degradation rate gradually decreased and the curves tended to be flat after reaching the peak loading. In general, the secant stiffness gradually decreased with the increase in displacement, where the degradation was initially fast, followed by a gradual slowing down in order to become stable. When compared to the specimens with lesser (*k*_m_), it was noticed that the stiffness curve of the specimen with the larger (*k*_m_) was superior.

## 5. Parameter Sensitivity Analysis

By quantifying the data of various factors and conducting a grey correlation analysis, the aim is to obtain each factor’s influence and relevance degree on the main behavior. It is an analysis method that effectively distinguishes the main and secondary factors [29]. Therefore, the effect of different factors on the seismic behavior of the S-RACFST frame can be compared through this method. Additionally, the degree of influence can also be used to judge when conflicts arise. In order to obtain the effect law of various factors on the seismic behavior for the S-RACFST frame, a grey correlation analysis was conducted on the frame’s ductility coefficient and energy dissipation capacity.

### 5.1. Determining the Standard Classification Sequence

Based on our previous test findings, in this study, the S-RACFST frame ductility coefficient and energy dissipation capacity of the test value [23] were selected as the reference sequence *X*_0_(*k*). Additionally, different variation factors, including the (*n*), (*i*), and (*k*_m_) were used as the comparison sequence *X_m_*(*k*), where *k* = 1,2,…, *p* and *m* = 1,2,…, and *q*. Then, all the values were extracted and relisted as comparable sequences.

(11) $$\begin{array}{l} X_{0}=X_{0}(1),X_{0}(2),......,X_{0}(p)\\ X_{1}=X_{1}(1),X_{1}(2),......,X_{1}(p)\\ ......\\ X_{q}=X_{q}(1),X_{q}(2),......,X_{q}(p) \end{array}$$

### 5.2. Normalization and Grey Correlation Coefficient

The method of non-dimension for the reference sequence and comparison sequence, in order to reduce the influence of parameter dimensions and numerical fluctuations, was selected. In this study, the normalization method, as formulated in Equation (12), was adopted.

(12) $$x_{m}(k)=\frac{X_{m}(k)}{\frac{1}{p}\sum_{m=1}^{p}X_{m}(k)}$$

The calculation method, with respect to the grey correlation coefficient ξ*_m_*, adopts the formula reported by Tian et al. [29], as shown in Equations (13)–(16).

(13) $$\xi_{m}\left[x_{0}(k),x_{m}(k)\right]=\frac{\underset{m=1,p}{\min}\;\underset{k=1,q}{\min}\Delta_{m}(k)+\rho \underset{m=1,p}{\max}\;\underset{k=1,q}{\max}\Delta_{m}(k)}{\Delta i+\rho \underset{m=1,p}{\max}\;\underset{k=1,q}{\max}\Delta i(k)}$$

(14) $$\Delta_{m}(k)=\left|x_{0}(k)-x_{m}(k)\right|$$

(15) $$\underset{m=1,p}{\min}\;\underset{k=1,q}{\min}\Delta_{m}(k)=\underset{m}{\max}(\underset{k}{\max}\left|x_{0}(k)-x_{m}(k)\right|)$$

(16) $$\underset{m=1,p}{\max}\;\underset{k=1,q}{\max}\Delta_{m}(k)=\underset{m}{\min}(\underset{k}{\min}\left|x_{0}(k)-x_{m}(k)\right|)$$

where *ρ* is the grey correlation coefficient, *ρ*∈[0, 1], and *ρ* is usually given as 0.5 [29].

### 5.3. Grey Correlation Degree and Sensitivity Assessment

**Figure 13:**
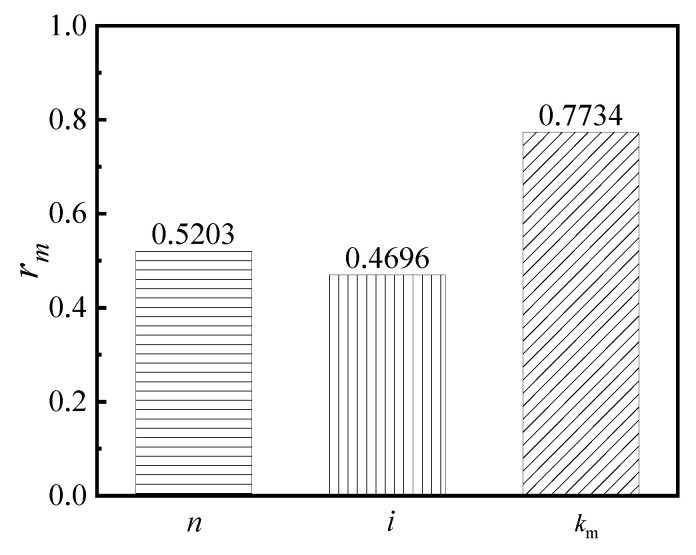
The grey correlation degree between the ductility coefficient and the other varying factors.

**Figure 14:**
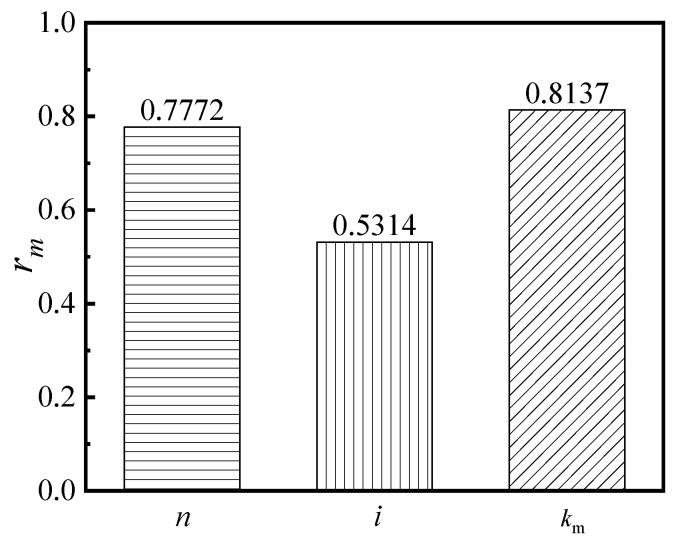
The grey correlation degree between the energy dissipation capacity and the other varying factors.

The grey correlation degree (*r_m_*), which was measured as the correlation strength between the two variables, indicates how closely the comparison sequence and the reference sequence are related. Therefore, by calculating the grey correlation degree *r_m_*, one can evaluate the different sequence levels. When the *r_m_* approached 1.0, the correlation between the reference sequence and the comparison sequence was found to be stronger. In addition, the grey correlation degree *r_m_* was shown in Equation (17), whereby the correlation degree between the ductility coefficient and the varying factors are shown in Figure 13. The correlation degree between the energy dissipation capacity and the varying factors is shown in Figure 14.

(17) $$r_{m}=\frac{1}{p}\sum_{m=1}^{n}\xi_{m}\left[x_{0}(k),x_{m}(k)\right]$$

As shown in Figure 13, the correlation degree of the varying factors with respect to the ductility coefficient is ordered, as follows: (*k*_m_) > (*n*) > (*i*). Additionally, the results with respect to this are also as follows: (1) The (*k*_m_) possesses a significant correlation with the ductility coefficient of the S-RACFST frame, and the *r_m_* is as high as 0.7734; (2) the (*n*) and the (*i*) possess a secondary correlation with the ductility coefficient of the S-RACFST frame, whereby the values of *r_m_* are 0.5203 and 0.4696, respectively. Furthermore, the influence of the (*n*) on the ductility coefficient of the S-RACFST frame is slightly more significant than the (*i*).

As shown in Figure 14, the correlation degree of the varying factors with respect to the energy dissipation capacity is ordered as follows: (*k*_m_) > (*n*) > (*i*). Additionally, the results with respect to this are as follows: (1) the (*k*_m_) and (*n*) possess a major correlation with the energy dissipation capacity of the S-RACFST frame, whereby the values of *r_m_* are 0.8137 and 0.7772, respectively, as well as being relatively close to each other; (2) the (*i*) possesses a secondary correlation with the energy dissipation capacity of the S-RACFST frame, and the correlation degree is relatively weak, whereby the *r_m_* value is 0.5314.

In summary, the (*k*_m_) and (*n*) possess a more significant impact on the seismic behavior of the S-RACFST frame. Moreover, the (*k*_m_) possesses a strong correlation with the ductility coefficient and energy dissipation capacity of the S-RACFST frame. As such, it is first recommended to consider the (*k*_m_) when designing the structure in order to ensure the seismic behavior of the RACFST frame. The design criterion of the “strong column and weak beam” of the RACFST frame can be realized by selecting an appropriate (*k*_m_), as well as further achieving a higher bearing and deformation capacity.

## 6. Conclusions

This study established a finite element model with respect to the seismic behavior for the S-RACFST frame specimen. The seismic behavior of the eight S-RACFST frame finite element specimens was analyzed within the various design parameters. The sensitivity of the various design parameters on the seismic behavior of the S-RACFST frame was revealed via grey correlation analysis. The conclusions were as follows: 

(1) The test curves agree with the finite element simulation curves of the S-RACFST frame that were developed by the ABAQUS software. The computational accuracy can meet the requirements, as well as address the response of the S-RACFST frame, which is effectively simulated under the seismic effects. Thus, the finite element model developed in this study is feasible with respect to the seismic behavior analysis of the S-RACFST frame. 

(2) With the axial compression ratio increasing from 0.2 to 0.4, the hysteretic curve of the specimen becomes fuller, the horizontal bearing capacity decreases gradually, and the displacement ductility coefficient increases by 28.5%. At the end of the loading phase, the equivalent viscous damping coefficient of 0.348 is obtained with respect to the specimens with an axial compression ratio of 0.4. The equivalent viscous damping coefficients of the specimen with the axial compression ratio of 0.4 were found to be 17.9% and 11.5% higher than that of the specimen with the axial compression ratio of 0.2 and the axial compression ratio of 0.3.

(3) With an increase in the beam–column line stiffness ratio, the hysteretic curves of the specimens show a full fusiform shape, and, subsequently, the load capacity gradually increases. At the end of the loading phase, all of the specimens’ equivalent viscous damping coefficients were found to be higher than those of the traditional reinforced concrete structures. The change in the displacement ductility coefficient was found to increase at first, then decrease. When the beam–column line stiffness ratio was at 0.41, a higher ductility within the range of changing parameters was found. The reason for this is due to the fact that the appropriate beam–column line stiffness ratio causes the beam to obtain a better transfer horizontal force, such that the two columns work well together; however, when it is too large, it will lead to a ductility reduction. Therefore, when the beam–column line stiffness ratio is appropriate, the frame can demonstrate good energy dissipation capacity.

(4) With the increase in the yield bending moment ratio of the beam–column from 0.10 to 0.31: the hysteretic curves of the specimens become fuller and fuller; the peak bearing capacities of the specimens increase; and the positive peak load and negative peak load increase by 16.4% and 22.8%, respectively. The displacement ductility coefficients were found to be close to three, thereby demonstrating good seismic performance. The stiffness curve of the specimen with a large yield bending moment ratio with respect to the beam–column was found to be significantly higher than that of the specimen with a small ratio. In addition, each specimen exhibited a great energy dissipation capacity. Furthermore, the results show that the variation in the yield bending moment ratio of the beam–column possesses a significant influence on the bearing capacity of the frame, such that the design requirements of the bearing capacity can be realized by designing the yield moment ratio of the beam–column in regard to engineering practice.

(5) According to the parameter sensitivity analysis, the beam–column yield bending moment ratio significantly influences the seismic behavior of the S-RACFST frame. It is recommended that the yield bending moment ratio of the beam–column be considered first in order to ensure the seismic behavior of the RACFST frame. Moreover, a higher load-bearing and deformation capacity can be achieved by selecting an appropriate yield bending moment ratio of the beam–column.

(6) This study shows that the S-RACFST frames possess an excellent seismic performance within the range of design parameters. The data parameters provided in this paper can be referred to when designing such frames in earthquake areas, thereby providing a reference for engineering designs.

The finite element model of the single-story and single-span structures established in this study is based on previous experiments, which can reflect the stress state of the actual structure to a certain extent. The single-story and single-span frame form is the most basic component unit. As such, the multi-story building frame can be regarded as the combination and superposition of single-story and single-span frames. Therefore, the experimental design of multi-story buildings can refer to the results of this study. After that, the hysteretic performance experiment of the multi-story building frame can be carried out. When such an effort is conducted, the finite element model of the multi-story building frame can then be established. The mechanical characteristics and failure laws of the multi-story building can be understanding.

## Figures and Tables

**Table 1:** Physical properties of the recycled aggregate.

Grading (mm)	Bulk Density (kg/m^3^)	Apparent Density (kg/m^3^)	Water Absorption (%)	Crush Index (%)
5–25	1382	2476	6.55	12.61

**Table 2:** Key parameters of the concrete damage plasticity model.

Model Parameters	Dilation Angle	Eccentricity	*f*_b0_/*f*_c0_	*K*	Viscosity Parameter
Values	30°	0.1	1.16	0.667	0.0005

**Table 3:** The simulation and test regarding the characteristic value of the peak point of the backbone curve.

Loading Direction	Test		Simulation		Comparison	
	Δ_p_^T^ (mm)	*P*_p_^T^ (kN)	Δ_p_^S^ (mm)	*P*_p_^S^ (kN)	Δ_p_^S^/Δ_p_^T^	*P*_p_^S^/*P*_p_^T^
Positive	24.02	190.53	18.00	207.65	0.749	1.090
Negative	−29.90	−174.25	−18.00	−171.54	0.602	0.984

Note: Δ_p_^T^ represents the displacement of the peak point of the test curve; *P*_p_^T^ represents the peak bearing capacity of the test curve; Δ_p_^S^ represents the displacement of the peak point of the simulation curve; and *P*_p_^S^ represents the peak bearing capacity of the simulated curve.

**Table 4:** Model parameter of the S-RACFST frame finite element specimen.

Number	Axial Compression Ratio (*n*)	Beam-Column Line Stiffness Ratio (*i*)	Yield Bending Moment Ratio of Beam-Column (*k*_m_)
N1-I2-K2	0.36	0.41	0.19
N2-I2-K2	0.20	0.41	0.19
N3-I2-K2	0.30	0.41	0.19
N4-I2-K2	0.40	0.41	0.19
N1-I1-K2	0.36	0.62	0.19
N1-I3-K2	0.36	0.31	0.19
N1-I2-K1	0.36	0.41	0.10
N1-I2-K3	0.36	0.41	0.31

Note: *n* = *N*/(*f*_ck_*A*_c_ + *f*_y_*A*_s_); *N* is the axial force; *f*_ck_ is the RAC axial compressive strength; *A*_c_ represents the sectional area of the compressed concrete; *f*_y_ represents the measured yield strength of steel; and *A*_s_ is the sectional area of the steel tube. *i* = *i*_b_/*i*_c_ = *I*_b_*h*/*I*_c_*l*, *I*_b_ represents the inertia moment of the beam section; *l* represents the calculation span of the beam; *I*_c_ represents the inertia moment of the column section; and *h* represents the calculation height of the frame column. *k*_m_ = *M*_by_/*M*_cy_, $M_{by}=f_{yk}A_{s}^{a}(h_{0}-a_{s}^{\prime })$, $M_{by}=f_{yk}A_{s}^{a}(h_{0}-a_{s}^{\prime })+0.5N_{G}h(1-N_{G}/f_{ck}bh)$, $A_{s}^{a}$, and *f*_yk_ are the cross-sectional area and the strength standard value for longitudinal tensile steel bars, respectively. Moreover, *h*_0_ represents the virtual height for the component section; $a_{s}^{\prime }$ represents the distance from the resultant point of longitudinally compressed steel bars to the near side of the section; and *b* and *h* represent the width and height of the rectangular section of the component, respectively.

**Table 5:** The displacement ductility coefficient of specimens under different (*n*).

Number	Loading Direction	Yield Point		Peak Point		Failure Point		*μ =* Δ_u_*/*Δ_y_
		*P*_y_ (kN)	Δ_y_ (mm)	*P*_m_ (kN)	Δ_m_ (mm)	*P*_u_ (kN)	Δ_u_ (mm)	
N2-I2-K2	Positive	205.68	11.26	241.97	18.00	210.70	36.00	3.20
	Negative	156.47	14.05	190.22	24.00	168.02	36.00	2.56
	Average	181.07	12.66	216.10	21.00	189.36	36.00	2.88
N3-I2-K2	Positive	189.98	10.27	223.52	18.00	199.80	36.00	3.51
	Negative	148.13	12.81	182.22	18.00	158.88	36.00	2.81
	Average	169.06	11.54	204.37	18.00	179.34	36.00	3.16
N4-I2-K2	Positive	168.60	9.29	198.36	12.00	174.42	36.00	3.88
	Negative	128.61	10.24	159.40	18.00	137.73	36.00	3.52
	Average	148.61	9.77	178.88	15.00	156.07	36.00	3.70

Note: Δ_y_ and *P*_y_ represent displacement and load at the yield point, respectively; Δ_m_ and *P*_m_ represent the displacement and load at the peak point, respectively; and Δ_u_ and *P*_u,,_ respectively, stand for the displacement and load at the failure point.

**Table 6:** Displacement ductility coefficient of the specimens with different (*i*).

Number	Loading Direction	Yield Point		Peak Point		Failure Point		*μ =* Δ_u_*/*Δ_y_
		*P*_y_ (kN)	Δ_y_ (mm)	*P*_m_ (kN)	Δ_m_ (mm)	*P*_u_ (kN)	Δ_u_ (mm)	
N1-I1-K2	Positive	182.99	10.60	215.29	18.00	182.99	31.42	2.96
	Negative	150.97	12.04	177.22	18.00	150.64	29.32	2.43
	Average	166.98	11.32	196.26	18.00	166.82	30.37	2.70
N1-I2-K2	Positive	180.96	9.96	207.65	18.00	185.62	36.00	3.61
	Negative	120.41	11.38	171.45	18.00	147.36	36.00	3.16
	Average	150.69	10.67	189.60	18.00	166.49	36.00	3.39
N1-I3-K2	Positive	168.22	11.28	198.40	18.00	168.64	32.88	2.91
	Negative	138.00	11.40	163.75	18.00	139.18	29.75	2.61
	Average	152.56	11.38	181.08	18.00	153.91	31.26	2.76

**Table 7:** The displacement ductility coefficient of specimens with different (*k*_m_).

Number	Loading Direction	Yield Point		Peak Point		Failure Point		*μ =* Δ_u_*/*Δ_y_
		*P*_y_ (kN)	Δ_y_ (mm)	*P*_m_ (kN)	Δ_u_ (mm)	*P*_u_ (kN)	Δ_u_ (mm)	
N1-I2-K1	Positive	154.78	9.01	187.85	12.00	159.67	35.50	3.94
	Negative	113.18	10.53	148.73	18.00	126.42	32.71	3.11
	Average	133.98	9.77	168.29	15.00	143.05	34.11	3.52
N1-I2-K2	Positive	180.96	9.96	207.65	18.00	185.62	36.00	3.61
	Negative	120.41	11.38	171.54	18.00	147.36	36.00	3.16
	Average	150.69	10.67	189.60	18.00	166.49	36.00	3.39
N1-I2-K3	Positive	182.32	10.23	218.67	18.00	185.87	35.06	3.43
	Negative	150.11	11.50	182.59	18.00	155.20	34.59	3.01
	Average	166.21	10.87	200.63	18.00	170.54	34.82	3.22

**Table 8:** The *h*_e_ of specimens with different (*n*).

Number	Δ	2Δ	3Δ	4Δ	5Δ	6Δ
N2-I2-K2	0.044	0.07	0.091	0.143	0.227	0.295
N3-I2-K2	0.036	0.074	0.106	0.169	0.252	0.312
N4-I2-K2	0.034	0.086	0.145	0.226	0.291	0.348

**Table 9:** The *h*_e_ of characteristic point for specimens under different (*n*).

Number	Δ_y_	Δ_m_	Δ_u_
N2-I2-K2	0.123	0.147	0.295
N3-I2-K2	0.083	0.106	0.312
N4-I2-K2	0.108	0.157	0.348

**Table 10:** The *h*_e_ of specimens under different (*i*).

Number	Δ	2Δ	3Δ	4Δ	5Δ	6Δ
N1-I1-K2	0.03	0.09	0.163	0.269	0.356	0.381
N1-I2-K2	0.059	0.151	0.186	0.243	0.320	0.370
N1-I3-K2	0.026	0.071	0.142	0.228	0.321	0.354

**Table 11:** The *h*_e_ of characteristic points for specimens under different (*i*).

Number	Δ_y_	Δ_m_	Δ_u_
N1-I1-K2	0.082	0.163	0.279
N1-I2-K2	0.148	0.215	0.370
N1-I3-K2	0.067	0.142	0.268

**Table 12:** The *h*_e_ of specimens under different (*k*_m_).

Number	Δ	2Δ	3Δ	4Δ	5Δ	6Δ
N1-I2-K1	0.035	0.137	0.152	0.214	0.295	0.353
N1-I2-K2	0.059	0.151	0.186	0.243	0.320	0.370
N1-I2-K3	0.034	0.084	0.120	0.177	0.238	0.273

**Table 13:** The *h*_e_ of characteristic points for specimens under different (*k*_m_).

Number	Δ_y_	Δ_m_	Δ_u_
N1-I2-K1	0.116	0.165	0.363
N1-I2-K2	0.148	0.215	0.370
N1-I2-K3	0.096	0.120	0.326

## Data Availability

The data used to support the findings of this study are available from the corresponding author on reasonable request.
